# Incidence and temporal distribution of SARS-CoV-2 circulating in the municipal wastewaters of the Buffalo City region, Eastern Cape, South Africa

**DOI:** 10.1016/j.gloepi.2025.100229

**Published:** 2025-11-11

**Authors:** Okuhle Mayoyo, Luyanda Msolo, Kingsley E. Ebomah, Nolonwabo Nontongana, Anthony I. Okoh

**Affiliations:** a SAMRC Microbial Water Quality Monitoring Centre, University of Fort Hare, Alice 5700, South Africa; b Applied and Environmental Microbiology Research Group (AEMREG), Department of Biotechnology and Biological Sciences, University of Fort Hare, Alice 5700, South Africa

**Keywords:** Influent, Municipal wastewater, RT-qPCR, SARS-CoV-2, Wastewater surveillance

## Abstract

**Background:**

The global health catastrophe of the Coronavirus Disease 2019 (COVID-19) resulted from the emergence and proliferation of a *Betacoronavirus*, the Severe Acute Respiratory Syndrome Coronavirus 2 (SARS-CoV-2). Since the unprecedented pandemic, over 6 million fatalities associated with COVID-19 have been observed on a global scale, and the world economy has taken a toll. The virus is discharged into the wastewater milieu, posing significant environmental and public health concerns. Hence, this investigation sought to explicate the incidence of SARS-CoV-2 RNA in the municipal wastewater of Buffalo City Metropolitan Municipality (BCMM).

**Methods:**

Raw wastewater samples were obtained from inlets of various municipal wastewater treatment plants (WWTPs) in BCMM for a 46-week surveillance period. Commercial kits were utilized to extract viral Ribonucleic Acid (RNA) from 486 wastewater samples following the standard operating protocol. Subsequently, the Quantitative Reverse Transcription Polymerase Chain Reaction (RT-qPCR) was employed to profile the pervasiveness of SARS-CoV-2 RNA over the surveillance period.

**Results and conclusion:**

351 (72 %) possessed the nucleocapsid protein gene, signifying SARS-CoV-2 RNA in the municipal wastewater. A time series analysis revealed relatively higher SARS-CoV-2 RNA counts in autumn (2022). A moderate correlation (*r* = 0.43) was observed between the SARS-CoV-2 RNA and the estimated number of infected individuals. Nonetheless, the pervasiveness of SARS-CoV-2 in this environment is of critical public health significance, offering a valuable indicator of community-level viral spread. The present study's findings substantiate our understanding that wastewater possesses pathogens of interest, emphasize the importance of monitoring wastewater.

## Background

Wastewater is a non-invasive medium to monitor communities for chemicals, pharmaceutical drug usage, and circulating pathogens. Due to the recent pandemic, wastewater surveillance rapidly transitioned from being relegated to a conventional approach [1,2]. Municipal wastewater influents are blueprints of agents circulating in respective communities.

Viruses are within the populace of microorganisms inhabiting the wastewater habitat. Viruses are naturally ubiquitous, and their prevalence in wastewater is unsurprising. Human excreta is among several sources of viruses, viral fragments, and genetic material in wastewater [3,4]. Additionally, infected individuals' virus-infected mucus/sputum or fluids infested with virus fragments/particles move through the gastrointestinal system into biological waste, reaching wastewater treatment facilities. Wastewater treatment facilities accumulate a variety of viruses excreted by individuals with apparent symptoms and those without [5]. Enteric viruses and the genetic material of some respiratory viruses are among the diverse categories ubiquitous in wastewater influents [[6], [7], [8]].

Detecting traces of pathogenic viruses in wastewater has helped predict viral outbreaks, which has been the case for SARS-CoV-2 [[9], [10], [11], [12]]. SARS-CoV-2 is the respiratory coronavirus driving the most recent pandemic. The Coronaviridae member primarily invades the respiratory system, and symptoms range from mild to severe disease and even death. The virus is accountable for over 6 million COVID-19-related fatalities globally [13]. The rapid identification and monitoring of this virus, a significant public health concern, is crucial in disease management practices. The pathogenic invasion by SARS-CoV-2 is concomitant with faecal shedding. Thus, determining the quantity of the coronavirus genetic material in wastewater can gauge the penetration and frequency of the coronavirus in communities [14].

Advancements in molecular techniques have facilitated the prompt determination of SARS-CoV-2 extracted from patient-derived specimens and environmental sources. This study extensively used the RT-qPCR for identifying and quantifying the coronavirus circulating in Buffalo City Metropolitan municipal wastewater samples. RT-qPCR is a molecular technique that reverse transcribes RNA into complementary DNA, amplifying it into numerous copies. The technique is quick, highly sensitive and selective. The data obtained from the reaction can be used to deduce the amount of the targeted gene in the test samples. Furthermore, coupling RT-qPCR and approximating the quantity of infected people provides a Wastewater-based Epidemiology (WBE) of SARS-CoV-2 RNA within the study area. Understanding and monitoring the circulating perilous pathogens is crucial. Such information can serve as a warning measure and is crucial for public health security.

There is diminutive data on the incidence and temporal dynamics of SARS-CoV-2 RNA in influent sourced from the designated wastewater treatment plants (WWTP) in BCMM in the Eastern Cape Province. Therefore, this research explicated the occurrence of SARS-CoV-2 RNA in municipal wastewater influent obtained from BCMM WWTPs. The study focused on analysing raw wastewater influent samples and applied the grab-sampling technique. According to Augusto et al. [15] this technique is preferred when evaluating the concentrations of substances, pathogens such as SARS-CoV-2, and pH levels, which may change rapidly and frequently. Additionally, the sampling areas were at distinct geographical locations. Therefore, the grab technique enabled us to cover all the sites, considering the massive distance, which would have made it impossible to manage collection from multiple points at time intervals. The data acquired from this study ought to serve as a surveillance system that can alert the possible public health security predicament prompted by SARS-CoV-2.

## Experimental design

### Ethical clearance certificate and permits

Ethical clearance and permission to commence the study were sought from and authorized by the University of Fort Hare Research Ethics Committee (UFH-REC) with ethics code OKO011SMAY01.

### Study area description

The study is based in BCMM in the Eastern Cape Province (ECP), which has 170,000 km^2^ of diverse landscape. It among South Africa's most impoverished provinces, mainly consisting of rural settlements with inadequate hygiene services and poor infrastructure [[16], [17], [18], [19], [20]], and it was the first to enter the country's second epidemic wave [21]. The Eastern Cape's population constitutes 13 % of South Africa, with an estimated population of 6.6 million [16]. The province is divided into six district municipalities and two metropolitan municipalities, one of which is BCMM, which has the second largest population density in the province [22] (317.8/km^2^), making it easier to disseminate SARS-CoV-2.

During the pandemic BCMM was one of the COVID-19 hotspots in South Africa [[23], [24], [25], [26]] and is a metropolitan area characterized by leisure activities, fine establishments, nature and gaming reserves, shopping centres and malls, and is home to several educational institutions [27,28]. People commute within and out of the municipal region for various reasons, such as work, education, and social activities, which consequently contribute to the spread of the coronavirus. The metropolitan nature of the municipality harbours a population from different socioeconomic standings.

Low-income individuals live in crowded and informal settlements located on the outskirts of BCMM, and some are part of essential services with increased risks of exposure to SARS-CoV-2. Some of these low-income communities are exposed to raw sewage. During the sudden surges of COVID-19, the healthcare services in the municipality were utterly overwhelmed, advancing the accelerated dissemination of the SARS-CoV-2 genome, delayed treatment, and increased mortality.

### Sampling sites

Study samples were sourced from certain WWTPs situated within BCMM of the Eastern Cape Province. Sites and samples collected from the wastewater treatment facilities were represented in codes respective to the collection points. Two influent collection points were identified in EB WWTP and QUIN WWTP, and samples from each point were coded aptly. The properties of each sample collection site are characterized in Table 1.

**Table 1:** Characteristics of the Buffalo City Metropolitan Municipality WWTPs. Two influent collection points were identified in EB WWTP (EB-A & EB-B) and QUIN WWTP (GON-A & GON-B).

WWT facility	Resource discharged into:	Wastewater technology used	System design capacity (ML/d)	Greendrop score (%) [29]	Population served by facility
BIS WWTP	Yellowwoods River	Dose processing and oxidation ponds	2	53	150,995
CEN WWTP	Buffalo River	Oxidation & Biofiltration	8	59	43,100
DIM WWTP	Mdizeni Stream (Tributary of Keiskamma River)	Activated sludge system	7	64	18,517
EB WWTP	Marine/sea discharge	Activated sludge system	40	73	141,000
KID WWTP	Mcantsi River	Oxidation ponds	0.4	38	69,900
MDA WWTP	Unknown tributary of Buffalo	Activated sludge system	24	63	112,900
QUIN WWTP	Sea discharge	Fine bubble aeration	18	63	30,400
REE WWTP	Buffalo River	Vertical aeration blade method Biological odour control system	10	50	25,400
SCH WWTP	Buffalo River	Activated sludge system Biofiltration, & Bioreactor system	7	60	35,000
ZWE WWTP	Buffalo River	Activated sludge system	9	58	48,900

### Sample acquisition and processing

About 500 mL samples of wastewater inlet were collected every week for 46-week surveillance period (June 2021–June 22) using grab-sampling technique (Table 1) which offers preliminary data on the wastewater components at the specific sampling time and conducting a temporal analysis. The wastewater was aseptically transferred into decontaminated 500 mL Nalgene bottles. The samples were kept at 4 °C to the National Wastewater Coronavirus Surveillance Laboratory situated at the SAMRC Microbial Water Monitoring Centre and processed at within 6 h of sampling.

The laboratory was decontaminated with ultraviolet (UV) light upon arrival. The wastewater samples were homogenized within a sterile lamina flow hood after decontamination. After that, aliquots of the samples were dispensed into 50 mL capacity centrifuge tubes (× 4 per sample, based on the sample's turbidity). The aliquots were then centrifuged at 2500 rpm for 20 min. After centrifuging, the supernatant was disposed of, and the concentrated pellet was utilized to extract nucleic acid and perform the subsequent RT-qPCR procedure.

### Total RNA extraction and quantification

The RNA extraction protocol optimized by Johnson et al. [30] was employed to extract total RNA from the processed wastewater influent samples. Approximately 7.5 mL of the pellet was used for the extraction of total RNA. The QIAGEN RNeasy PowerSoil Total RNA Kit (QIAGEN, Germany) was utilized to extract the total RNA in compliance with the manufacturer's guidelines. The Nanodrop™ One Microvolume UV–Vis spectrophotometer (Thermo Fisher Scientific, Waltham, USA) was used to validate the quantity and quality of the RNA.

Total nucleic acids with absorbance ratios between 1.8 – 2.0 at A260/A280 and A260/A230 wavelengths were selected for subsequent RT-qPCR and were stored at −80 °C. These samples were considered pure. Absorbance at 260/280 nm indicates how samples are from contaminating proteins as proteins absorb at 280 nm, a ratio lower than the abovementioned indicates high amounts of proteins and amino acids, relative to nucleic acids. Absorbance at 260/230 nm indicates the purity of samples from contaminants such as phenol, guanidine hydrochloride and salts that may absorb at 230 nm.

### Profiling the presence of SARS-CoV-2 by RT-qPCR

The detection and quantification of SARS-CoV-2 RNA was done by targeting the Nucleocapsid protein-encoding gene (N) at SARS-CoV-2_N1-P and SARS-CoV-2_N2-P (2019-nCoV, CDC, EUA). This was achieved by utilizing the iTaq Probe-Based qPCR assays (BIO-RAD, USA) and the QuantStudio 5 qPCR system (Applied Biosystems). Table 2 describes the probes applied to detect the coronavirus in the wastewater.

**Table 2:** 2019 nCoV N1 and N2 oligonucleotide primers/probes applied to determine SARS-CoV-2 RNA in the municipal wastewater.

Name	Description	Oligonucleotide sequence (5′- 3′)	Reference
SARS-CoV-2_N1-P	2019 nCoV_N1 Primer/ Probe	F 5′-GAC CCC AAA ATC AGC GAA AT-3′ R 5′-TCT GGT TAC TGC CAG TTG AAT CTG-3′ Probe FAM-ACC CCG CAT TAC GTT TGG ACC-NFQ-MGB	[30,31]
SARS-CoV-2_N2-P	2019 nCoV_N2 Primer/ Probe	F 5′-TTA CAA ACA TTG GCC GCA AA-3′ R 5′-GCG CGA CAT TCC GAA GAA-3′ Probe FAM-ACA ATT TGC CCC CAG CGC TTC AG-NFQ-MGB	

A MicroAmp™ Optical 96-Well reaction plate (Applied biosystems®) was for the RT-qPCR. A 10-fold dilution was used to aseptically prepare a working stock of total RNA to optimize the concentration. 1 uL of pure nucleic acids was aliquoted into 9 uL of RT-qPCR reaction mixture. The reaction mixture comprised of 5 uL iTaq universal probes reaction mix, 0.25 uL of iScript RT advanced reverse transcriptase (BIORAD, USA), 0.5 uL of each SARS-CoV-2_N1-P and SARS-CoV-2_N2-P and 3.25 uL of aseptic Nuclease free water (BIO-RAD, USA), summing up to 10 uL per reaction. The reaction plate was sealed with adhesive covers under aseptic conditions. The reaction mixture was spun down for 10 min at 500 rpm on a plate shaker. The plate was then inserted into a QuantStudio 5 qPCR system (Applied biosystems), and the cycling conditions in Table 3 were set.

**Table 3:** The temperature and time parameter settings for target gene amplification using specific N primer/probes that target the coronavirus's nucleocapsid region.

		AMPLIFICATION				
Real-time PCR system	Setting	Reverse transcription reaction	Polymerase activation and DNA denaturation	Denaturation at 95 °C	Annealing/Extension + Plate read at 60 °C	Cycles
Quantstudio 5 PCR machine	Fast/Standard	10 min at 95 °C	3 min at 95 °C	15 s at 95 °C	1 min at 60 °C	40

The qPCR processes occurred in 40 cycles (Table 3). During the amplification of the cDNA, fluorescent labelling allowed the collection of amplification data. The Design & Analysis 2.4 RT PCR Software (Applied biosystems®) generated amplification plots and standard curves to monitor and measure the efficiency of the reaction. Table 2, Table 3 outline the probes and the PCR cycling conditions used to ascertain and enumerate SARS-CoV-2 genomes.

To obtain the measurable data on all wells, efficiency of the reaction was determined though the analysis of RT-qPCR parameters which further guide the quality of the amplicons. For quality assurance and accuracy, the PCR efficiency must be between 90 and 100 % which corresponds to a slope range of −3.6 to −3.3, the correlation values as close to 1 as possible, the standard error which must be as close to 0 as possible, a Cycle Threshold (C_T_) (≤ 35 cycles as C_T_ values) determine the positive samples at the detection threshold of 0.02 per reaction [30,32].

To evaluate the overall concentration of the SARS-CoV-2 RNA, the figures deduced (average quantity of genome copies (GC)) from RT-qPCR amplification were used, and the values were substituted into Eq. (1) and Eq. (2).

(1) $$\text{N1 and N2 average quantity}\times \text{dilution factor}=\text{SARS}-\mathrm{CoV}-2\;\mathrm{RNA}\;\text{concentration}\;\left(\mathrm{GC}/\mathrm{\mu L}\right)$$

(2) $$\text{SARS}-\mathrm{CoV}-2\;\mathrm{RNA}\;\left(\mathrm{GC}/\mathrm{mL}\right)=\text{SARS}-\mathrm{CoV}-2\;\mathrm{RNA}\;\text{concentration}\;\left(\mathrm{GC}/\mathrm{\mu L}\right)\times 10^{3}/\text{sample volume}\;\left(\mathrm{mL}\right)$$

### WBE to estimate the average quantity of people infected in the study area throughout the surveillance period

The protocol detailed in a study by Hadi et al. [33] was employed to estimate the infection prevalence (%) and the average number of people contributing to the frequency of SARS-CoV-2 RNA in the wastewater obtained from the selected municipal wastewater treatment facilities. The average daily stool mass per capita per day is 196 g per person, the average shedding rate ranging from 10^2.56^ to 10^7.67^ gene copies per gram of faeces, the total influent flowing into the facility (L/d) (lack of data on the average influent flowing into the inlet daily) and the population served by each facility were incorporated into Eq. (3) [34] to estimate the number of infected persons and Eq. (4) to estimate infection prevalence in the designated populations of Buffalo City.

(3) $$\text{Persons infected}=\left[\left(\mathrm{RNA}\;\text{copies}/\text{Litres of wastewater}\right)\times \left(\text{Litres of wastewater}/\mathrm{day}\right)\right]/\left[\left(g\;\text{faeces}/\text{person}–\mathrm{day}\right)\times \left(\mathrm{RNA}\;\text{copies}/g\;\text{faeces}\right)\right]$$

(4) $$\text{Estimated infection prevalence}\;\left(\%\right)=\left(\text{Persons infected}/\text{Population served}\right)\times 100$$

## Results and discussion

Wastewater is an intricate medium comprising organic and inorganic substances, an array of microorganisms, fragments of genetic material and viruses [35,36]. A spectrum of microorganisms, including viruses, are pervasive and obstinate in treated and untreated wastewater and their catchment water bodies [5,37]. Numerous investigations have reported viruses in municipal wastewater, and their findings ascertain the evidence of viral genomes in the wastewater milieu [7,[38], [39], [40]].

### Ribonucleic acid concentrations detected from the municipal wastewater retrieved from selected BCMM WWTPs

This report presents RNA concentrations without normalization because of the absence of a readily obtainable flow rate across all WWTP during the surveillance period. A total of 486 samples were retrieved from the wastewater treatment facilities. On average, influent samples collected from site EB-A yielded the highest average RNA concentration of 1.36 × 10^3^ ng/μL, and the lowest concentration was observed in wastewater influent samples retrieved from REE WWTP. Analysis of all sites revealed a median concentration of 1.08 × 10^3^ ng/μL, similar to works by Wu et al. [41] and Foladori et al. [42] about an enumerated viral load of SARS-CoV-2 in wastewater. Nevertheless, the same protocol employed by Tomasino et al. [43] yielded a total RNA concentration of approximately 0.04 × 10^3^ ng/μL. This variation accounts for innumerable factors, including population dynamics, the organic load, dilution and wastewater influent flow rate [44,45]. Fundamentally, the variations in nucleic acid concentrations in wastewater are inevitable.

Wastewater influent samples from Buffalo City Metropolitan wastewater treatment facilities yielded average total RNA concentrations ranging between 0.8 × 10^3^ – 1.5 × 10^3^ ng/μL. The EB-A sampling site yielded an overall higher average total RNA concentration of 1.36 × 10^3^ ng/μL. Samples from this site were observed as highly turbid with a moderate flow throughout the study period, and the EB drainage reticulation served four suburbs with a combined population, as shown in Table 1. The incidence of RNA in the wastewater samples confirms the real-time viral indications in the wastewater environment [[46], [47], [48]].

Pure nucleic acids are utilized to screen the nucleocapsid gene loci molecularly. The nucleocapsid protein is fundamental for encapsulating the viral genome into the ribonucleoprotein complex and virion formation. It is the predominant protein in virions and is a potential target for most therapeutic practices [49,50]. It is a significant diagnostic indicator for COVID-19 as the nucleocapsid-inducing gene is exclusive to SARS-CoV-2 and indicates the pathogen's occurrence in test samples.

Average values of −3.6 and −3.4 of the subsequent standard curve slope for N1 and N2, respectively, were observed from the qPCR. The average values observed for the amplification efficiency were 90.5 % and 95.6 %, which were well within the recommended range from 90 % to 105 % [6,30]. Furthermore, the correlation values (R^2^) met the requirement of being as close to 1 as possible; on average, R^2^ values of 0.998 and 0.963 for N1 and N2, respectively. The standard error (close to 0) for targets N1 and N2 were 0.08 and 0.30, respectively, indicating an efficient reaction according to Johson et al. [30]. These parameters validated the prominent reaction sensitivity, efficiency and quality of the amplicons.

Moreover, a data sheet was exported to evaluate the metrical data, as it was crucial in determining the quality of the amplicons, and consequently, the target was quantified.

### SARS-CoV-2 RNA obtained from the municipal wastewaters of selected BCMM WWTPs

A total of 351 (72 %) samples out of 486 samples possessed the N1, N2 PCR loci of the N gene, implicative of SARS-CoV-2; therefore, SARS-CoV-2 RNA was detected in the municipal wastewaters of BCMM. Table 4 encapsulates the proportional allocation of the coronavirus in the municipal wastewater recovered from each site.

**Table 4:** Incidence profiles of the SARS-CoV-2 RNA observed in the municipal wastewater obtained from the selected WWTPs.

Sampling point (WWTP)	Number of samples retrieved from the sites	Samples positive for SARS-CoV-2 genome (%)
BIS	28	86
CEN	39	72
DIM	28	75
EB-A	35	69
EB-B	45	76
GON-A	45	80
GON-B	45	67
KID	45	56
MDA	46	59
REE	38	71
SCH	46	72
ZWE	46	74

The incidence of SARS-CoV-2 RNA in the municipal wastewater samples signified the occurrence of COVID-19 in the region. Most of the samples retrieved from BIS WWTP possessed the nucleocapsid protein gene PCR loci (N1 and N2), indicative of SARS-CoV-2. The average concentrations for N1 and N2 were used for quantifying SARS-CoV-2 RNA as several studies have demonstrated positive correlations between the two [51,52]. Parameters including mean, standard deviation, standard error, confidence level under 95 %, confidence level over 95 %, minimum value and maximum value were considered for the statistical analysis as shown in Table 5. Variability was observed throughout suggesting a skewed distribution. This is attributed to the frequent spikes throughout the surveillance period. (See Table 6.)

**Table 5:** Overall descriptive statistics for the SARS-CoV-2 RNA concentrations in wastewater.

Sampling point	Mean	SD	SE	LB	UB	Min	Max
BIS	1764.58	3224.17	569.96	647.47	2881.70	0	17,602.09
CEN	1246.37	2603.67	411.68	439.48	2053.25	0	13,848.77
DIM	1374.84	1733.22	327.55	732.84	2016.83	0	6250.90
EB-A	1834.27	3417.32	569.55	717.94	2950.59	0	16,661.75
EB-B	2309.72	5082.15	766.16	808.04	3811.40	0	24,205.98
GON-A	1499.07	2725.50	410.88	693.73	2304.40	0	10,667.08
GON-B	885.59	1477.29	222.71	449.07	1322.10	0	6667.40
KID	1435.90	4426.06	652.59	156.83	2714.97	0	26,905.29
MDA	969.80	1657.42	244.37	490.83	1448.77	0	6145.62
REE	1992.34	4126.42	669.39	680.33	3304.35	0	17,368.05
SCH	664.12	1114.12	164.27	342.15	986.08	0	5505.48
ZWE	1174.93	1715.08	252.88	679.29	1670.56	0	7095.53

**Table 6:** Estimated number of infected persons and the estimated prevalence of infection (%) potentially contributing to the levels of SARS-CoV-2 RNA detected in the wastewater from the designated sites across the study period.

WWTP	Capacity (L/d)	Average SARS-CoV-2RNA (GC/L)	Population served	No. persons infected (Average)	Prevalence of infection (%)
BIS	2,000,000	1764,6	150,995	4	0,007
CEN	8,000,000	1246,4	43,100	3	0,005
DIM	7,000,000	1374,8	18,517	3	0,006
EB	40,000,000	1911,3	141,000	4	0,008
KID	400,000	1435,9	69,900	3	0,006
MDA	24,000,000	969,8	112,900	2	0,004
QUIN	18,000,000	1269,5	30,400	3	0,005
REE	10,000,000	1992,3	25,400	4	0,008
SCH	7,000,000	664,1	35,000	1	0,003
ZWE	9,000,000	1174,9	48,900	2	0,005

On observation, influent samples retrieved from the BIS WWTP ranged from clear to slightly turbid throughout the surveillance period. The average viral load observed in the wastewater from this facility is 1.76 × 10^3^ GC/mL. However, the sampling site EB-B yielded the highest SARS-CoV-2 signals on average. On observation, wastewater-influent samples collected from the EB WWTP were extremely turbid throughout the surveillance period. The average viral load observed in the wastewater of this facility is 1.83 × 10^3^ GC/mL for EB-A and 2.30 × 10^3^ GC/mL for EB-B.

**Fig. 1:**
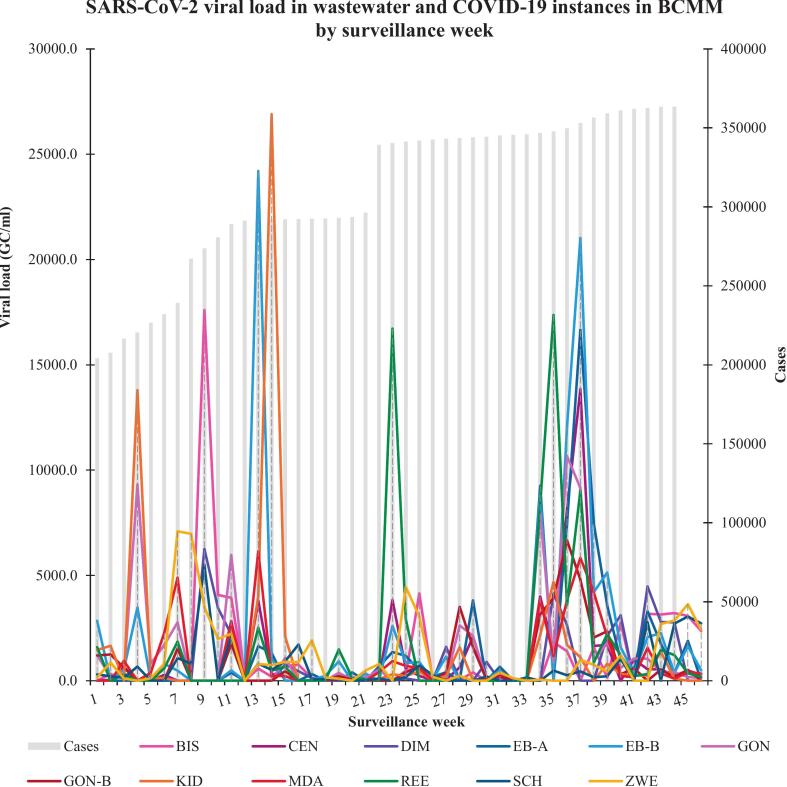
Chronological inclinations of the SARS-CoV-2 observed in the wastewater acquired from municipal WWTPs. The light grey bars on the secondary axis indicate the lab-confirmed clinical cases reported in the municipal region (NICD, 2021, 2022). A rapid rise in COVID-19 cases was reported from epidemiological week 22 to epidemiological week 46, i.e. from 02nd December 2021 to 30th June 2022, followed by a similar trend of the SARS-CoV-2 RNA in wastewater. Moreover, significant spikes in the SARS-CoV-2 wastewater signals are observed within that period (EPI week 34 (07th April 2022) to EPI week 38 (05th May 2022)).

A temporal analysis was constructed to deduce the chronological fluctuations of the SARS-CoV-2 RNA and compare the trends with clinical case data (extracted from the National Institute for Communicable Diseases of South Africa (NICD) surveillance reports) across the Buffalo City municipal region over 46 weeks. Variations in SARS-CoV-2 signals in the municipal wastewater were observed throughout the study period, similar to works by Street et al. [53], Shrestha et al. [54] and Qongwe et al. [55]. This observation was expected as COVID-19 levels cumulated nationwide [56,57] and internationally [58]. The municipal wastewater retrieved from the REE WWTP possessed an average viral load of 1.99 × 10^3^ GC/mL. Fig. 1 (below) reveals the temporal data of the SARS-CoV-2 signals detected in the municipal wastewater.

### Seasonal patterns of the SARS-CoV-2 RNA distribution across the BCMM municipal wastewaters

**Fig. 2:**
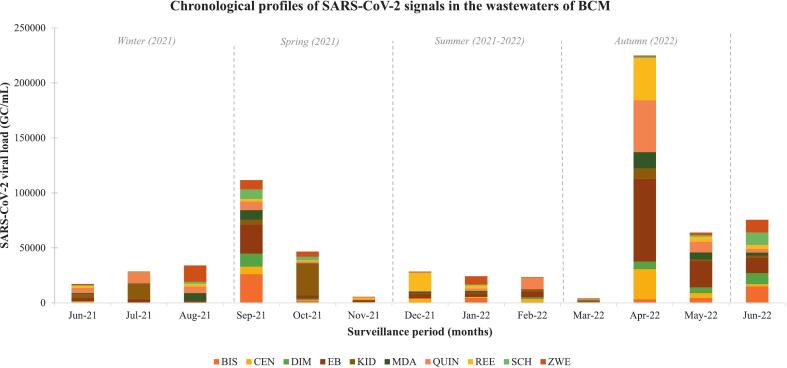
Chronological profiles of the SARS-CoV-2 wastewater signals across the selected WWTPs in BCM. Weekly data was consolidated into months. During the study, the winter surveillance period spanned from 24th June 2021 (beginning of sampling collection) to 3rd September 2021, spring spanned from 9th September 2021 to 25 November 2021, summer spanned from 2nd December 2021 to 24th February 2022 while autumn spanned from 3rd March 2022 to 26th May 2022. A rise in SARS-CoV-2 fragments in wastewater was observed during the winter (overlapping to early September 2021) and a spike was observed in the autumn surveillance period (prominent spikes in April 2022).

The temporal analysis reveals higher concentrations in winter, early spring, and mid-autumn (2022). Fig. 2 (below) illustrates the varied SARS-CoV-2 RNA trends throughout the seasons. The total viral load recovered across the study sites in winter was approximately 22.08 × 10^4^ GC/mL, spring 13.09 × 10^4^ GC/mL, summer 7.61 × 10^4^ GC/mL and autumn 29.30 × 10^4^ GC/mL*.* In essence, relatively high quantities of SARS-CoV-2 RNA were observed in the winter and autumn. The coronavirus is persistent at reduced temperatures and humidity, conditions often observed in the two seasons.

Winter and autumn are drier and characterized by lower temperatures. A study conducted in Canada highlighted that coronaviruses were among the numerous respiratory viruses that are accountable for seasonal epidemics, particularly in winter [59]. Seasonal changes influence biological ecosystems via environmental relations and alter infectious disease epidemiology. Climate change modulates host susceptibility and pathogen virulence. Several studies report increased mortality and morbidity in winter and autumn [60,61,62]. Elevated viral loads were noted in mid-autumn as temperatures declined and rainfall was scarce. The viral load in this season ranged from 3.80 × 10^3^–17.37 × 10^3^ GC/mL for other sites and 21.02 × 10^3^–810.99 × 10^3^ GC/mL from the EB WWTP. This site serves a larger population of 141,000 than other sites and is a combined sewer network.

Fig. 1, Fig. 2 illustrate the graphical depictions of SARS-CoV-2 wastewater signals' temporal patterns observed in the selected BCMM WWTPs over the 12-month sampling regime. The region was declared a COVID-19 hotspot between June 2020 and December 2020, exceeding Nelson Mandela Bay Metropolitan by 25 % (which is relatively larger in population). Higher concentrations (>5 × 10^3^ genomic copies/mL) can be observed from the fourth week (21st July 2021 to the 30th September 2021) of sample collection. This ensued during the third major epidemic of clinical cases. Variant Delta (B.1.617.2) predominated the third epidemic, which presented higher transmissibility than variant Beta (B.1.351, predominant in the second wave) due to spike protein mutations with an improved affinity for the ACE 2 receptors [63,64].

Samples were collected and analyzed during adjusted alert level 4, and sudden peaks were observed in surveillance week 4, approximated 4 days (21st July 2021) before the transition from adjusted alert level 4 to 3. According to the Network for Genomic Surveillance South Africa (NGS-SA), the Delta variant was detected in approximately 66 % of the specimens collected in the country while variants Alpha and Beta circulated at low frequencies in this period.

SARS-CoV-2 signals in wastewater rose again in weeks 7 to 11 (12th August 2021-16th September 2021) during the transition from adjusted alert levels 3 to 2. A significant rise was observed in surveillance week 13 (the day alert level 2 was lifted), and a spike in the BIS WWTP was detected in surveillance week 14 on 7th October 2021 (during the adjusted alert level 1). Supplementary Table S2 represents the estimated number of cases of infection throughout the study period and the SARS-CoV-2 lineages/variants contributing to disease dynamics and transmission, as per the Communicable Diseases Communique published by the NICD in 2021.

From surveillance weeks 14 to 22, the viral load stabilized. However, concentrations increased again for sites CEN, EB, MDA and REE in surveillance week 23 (9th December 2021), almost a week following the announcement of adjusted alert level 1, which was set in place on 1st December 2021, bringing about the fourth epidemic in the country predominated by Omicron (B.1.1.529). The circulating variant enhanced disease transmissibility [[65], [66], [67]], raising SARS-CoV-2 RNA levels in wastewater.

The viral load remained elevated until surveillance week 25 (27th January 2022). The upsurge in SARS-CoV-2 wastewater signals occurred throughout the local municipality elections, with an influx of people from different regions, and the country was at adjusted alert level one, where several restriction measures to prevent the transmission of the virus were lifted. A significant rise was observed in the REE WWTP with an average of 16.72 × 10^3^ GC/mL.

The plateau observed in REE between epidemiology weeks 25–33 represents the period when sample collection was utterly impossible due to damage sustained by the pump station from the heavy storms that had occurred in that period. However, the upward trend line is evidence of a surge in SARS-CoV-2 burden. The NICD also documented the sudden spike observed in surveillance week 23 (9th December 2021) in their WBE for the SARS-CoV-2 surveillance report released on 11th December 2021 [57]. A variant of concern was discovered in South Africa during this period, and the World Health Organization further classified it as variant Omicron (B.1.1.529). Omicron is highly transmissible, resists the efficacy of therapeutics, and partially escapes acquired immunity.

The chronological distribution of SARS-CoV-2 RNA in wastewater depicts similar trends to the increase in virus signals and the lifting of restriction measures. This can be attributed to basic human behaviour associated with freedom of movement. Environmental and climatic conditions may have facilitated the distribution of the viral fragments in the wastewater milieus.

### WBE to estimate the average number of people infected in the study area during the surveillance period

WBE is a laudable tool for the surveillance of emerging diseases via human excreta, thus assisting as a primary indicator of disease inclinations in various communities [68,69]. To estimate infection prevalence (%) and the average number of people contributing to the incidence of SARS-CoV-2 RNA in the wastewater retrieved from the selected municipal WWTPs, the protocol employed by Hadi et al. [33] was used.

The incidence of SARS-CoV-2 markers in wastewater is through infected individuals. The study demonstrates that the estimated number of individuals infected per day during the surveillance period was approximately 4 (0,007 %), 3 (0.005 %), 3 (0.006 %), 4 (0.008 %), 3 (0.006 %), 2 (0.004 %), 3 (0,005 %), 4 (0,008 %), 1 (0,003 %) and 2 (0,005 %) in the communities served by the BIS, CEN, DIM, EB, KID, MDA, QUIN, REE, SCH and ZWE WWTP, respectively. There is a paucity of WBE on the prevalence of SARS-CoV-2 RNA in wastewater in the Eastern Cape Province. Nevertheless, several studies nationally and globally have used the approach to detect the incidence of SARS-CoV-2 and variants in designated populations [10,[51], [52], [53],^70^].

Essentially, WBE estimated how much of the population served by each facility contributed to the obtained concentrations of SARS-CoV-2 RNA in wastewater at the time of sampling. In the study by Hadi et al. [33], the estimated number of individuals infected with SARS-CoV-2 ranged from 81 to 18,200 individuals. In contrast, the estimated number of infected individuals in this report ranged between 1 and 4 people. Parameters unique to each study area influenced these values. For instance, although the population served by each of the sites from both studies, was within the same range, the capacity of the plants affected the overall estimates. The capacity of the plants in this study ranged from 0.4 to 40 ML/d, while those in a study by Hadi et al. [33] ranged between 4.8 and 150 ML/d. Additionally, the estimated number of infected individuals and the prevalence of infection in the study area were affected by the lack of data on the average influent flowing into the inlet daily. As a result, the extremely low estimates limit the determination of the relationship between infection prevalence and the clinical reports in the region. Be as it may, the incidence of viral markers in wastewater is indicative of persistent infection and transmission in the study area.

On the other hand, some studies implemented WBE and observed that SARS-CoV-2 markers preceded clinical reports [10,[3], [4], [61], [62], [71], [72]]. There was a moderate correlation (*r* = 0.43) between the SARS-CoV-2 RNA average concentration detected in wastewater sourced from the selected WWTP in the Buffalo City region and the estimated number of infected individuals. The application of WBE in this study indicated the practicality of the approach to monitoring the population's infection status in conjunction with clinical data. WBE demonstrated its efficiency when used to monitor respiratory viruses in kindergarten [73] and to assess the seasonal distribution of the Dengue virus and the Chikungunya virus [74]. Table 7 provides an overview of the practical applications of WBE in different countries.

**Table 7:** Applications of wastewater-based surveillance for monitoring the presence of pathogens in wastewater matrices of different countries.

Country	Target pathogen	Detection Method	Summary of findings	Ref
Australia	SARS-CoV-2	RT-qPCR	Monte-Carlo simulations indicated a median range of 171 to 1090 infected persons	[8]
China	SARS-CoV-2	RT-qPCR	A significant correlation between SARS-CoV-2 markers in wastewater and daily reported cases	[75]
Ethiopia	SARS-CoV-2	RT-qPCR	94 % ww samples possessed SARS-CoV-2 RNA, estimated number of persons infected > COVID-19 cases reported	[71]
Italy	SARS-CoV-2	RT-qPCR	SARS-CoV-2 RNA detected in treated and untreated ww	[76]
Netherlands	SARS-CoV-2	RT-qPCR	SARS-CoV-2 wastewater signals were detected before the first detection of a clinical case	[72]
Nigeria	SARS-CoV-2, *Tuberculosis mycobaterium* & *Mycobacterium kansasii*	RT-qPCR, PCR	No samples possessed SARS-CoV-2 RNA, *M. fortitium* more prevalent (13, 32.5 %) than *M. kansasii* (11, 27.5 %)	[77]
Portugal	Dengue virus & Chikungunya virus	RT-PCR	DENV (in 25 % of ww samples) was more prevalent than CHIKV (11 % of ww samples)	[74]
South Africa	SARS-CoV-2	ddPCR	0–7.32 × 105 copies/100 mL observed in SARS-CoV-2 viral loads, stimated number of persons infected > COVID-19 cases reported	[10]
Spain	Monkeypox virus	RT-qPCR	Correspondings in MPXV signals in wastewater and clinical data	[83]
USA	SARS-CoV-2 and influenza A and B	RT-ddPCR	Detection of the respiratory viruses in manholes corresponded to clinical cases	[73]

Many factors that need to be studied, such as viral shedding, in-sewer factors, and population size, can influence SARS-CoV-2 RNA concentrations in influent wastewater [44]. For example, documented reports by Wu et al. [41] and Wilder et al. [78] reveal a direct correlation between the magnitude of the population and SARS-CoV-2 RNA concentrations in wastewater [41,79]. However, the magnitude of the population within the reticulation area should not be presumed to be stationary as people commute from one place to another for different reasons. Hence, Wade and colleagues explore the uncertainties and variabilities for wastewater monitoring and suggest factors such as the monitoring of instabilities of inhabitants within the catchment area to comprehend trends in RNA loads [44,80].

These findings confirm those of earlier studies, such as reviews and reports on the occurrences of SARS-CoV-2 RNA in the wastewater environment and the application of WBE as a surveillance tool for tracking the prevalence of diseases by authors such as Foladori et al. [42], La Rosa et al., Medema et al. [10], Ngqwala et al. [32] and Qongwe et al. [55]. Moreover, the overall approach applied in this study can provide a real-time warning for disease outbreaks and surveillance [81].

### Limitations

During the surveillance period, there were times when sites were utterly inaccessible for sample collection. Three sites (BIS, CEN, and DIM) were utterly inaccessible at times due to the absence of plant operators, and the plants were left locked and unattended. No wastewater streamed into the REE WWTP for 8 weeks, from 21st January 2021 to 17th March 2022. This is due to the damage sustained by the pump station caused by heavy storms. Sampling point EB-A was only discovered 9 weeks into sample collection. The WWTP personnel (EB, KID and QUIN) were absent for an apparent workshop between weeks 22 and 23, leaving the plants unattended and inaccessible. Furthermore, WBE calculations are crucial; however, without the appropriate parameters, such as the determined flow rate, estimating the quantity of infected individuals in the catchment area may not necessarily reflect the true epidemiology. A myriad of environmental and in-sewer factors can influence concentrations of viral signals detected in wastewater.

## Conclusion

These results emphasize the incidence of the ubiquitous nature of viruses in raw wastewater. The primary basis of SARS-CoV-2 fragments in this kind of environment is human excrement of symptomatic and asymptomatic carriers. Upon reviewing the data, the findings demonstrate the incidence of SARS-CoV-2 in the Buffalo City Metropolitan municipal wastewater. Higher levels of the viral fragments were recorded during outbreaks of SARS-CoV-2 variants throughout the epidemics and in the colder months (winter and autumn). Although seasons have no direct impact on the SARS-CoV-2 RNA concentrations in wastewater, reports suggest the impact of seasons on host susceptibility, pathogen dissemination rates, and the structure of the virus. Prominent spikes throughout the surveillance period could be attributed to the emergence of SARS-CoV-2 variants with varying degrees of transmissibility, mortality and morbidity. Nonetheless, this finding emphasizes the need to explore further the influence of season on the distribution of SARS-CoV-2. This can assist in preparing healthcare organizations and the public to better comprehend disease severity and circulation dynamics. A hasty public health response is imperative to avert further morbidity and mortality, as the pandemic has burdened healthcare systems in South Africa. Therefore, a regular wastewater surveillance study is highly recommended beyond this study period to envisage the probable resurgence of SARS-CoV-2 and other disease outbreaks. The findings of the present study corroborate the findings of numerous studies conducted elsewhere around the globe. Thus, it is imperative to develop proactive countermeasures for monitoring and managing pathogens of medical importance at community level and understand their genetic variations to alleviate potential outbreaks that the evolution of virulent factors may cause.

## CRediT authorship contribution statement

**Okuhle Mayoyo:** Writing – original draft, Visualization, Validation, Software, Methodology, Investigation, Formal analysis, Data curation, Conceptualization. **Luyanda Msolo:** Writing – review & editing, Validation, Supervision, Formal analysis, Conceptualization. **Kingsley E. Ebomah:** Writing – review & editing. **Nolonwabo Nontongana:** Project administration. **Anthony I. Okoh:** Writing – review & editing, Validation, Supervision, Resources, Project administration, Funding acquisition, Conceptualization.

## Funding

This research was made feasible by the financial support from the 10.13039/501100001322SAMRC http://www.samrc.ac.za/ (Grant number P790) and the 10.13039/501100001321National Research Foundation (NRF) (MND210622614695).

## Declaration of competing interest

The authors affirm no conflicting interests in this research.
