# Supplementary material for: Incidence and temporal distribution of SARS-CoV-2 circulating in the municipal wastewaters of the Buffalo City region, Eastern Cape, South Africa
Source: Glob Epidemiol. 2025 Nov 11;10:100229. doi: 10.1016/j.gloepi.2025.100229 (PMC12662084; doi:10.1016/j.gloepi.2025.100229)
Supplement: Supplementary material [file mmc1.docx]

**Supplementary data**

Supplementary Figure S1

Figure 3: A depiction of the SARS-CoV-2 fragments yielded by the wastewater recovered from each sampling site throughout the 46-week surveillance period. The proportion of influent samples exhibiting SARS-CoV-2 positivity is relatively higher (86%) in samples retrieved from the BIS WWTP, and a lower percentage (56%) is observed from samples collected from KID WWTP.

Supplementary Table S2

**Table 8:** COVID-19 epidemic periods by weekly incidence and estimated number of cases observed in the Eastern Cape Province.

| Epidemic | Peak date | Estimated number of cases | Weekly cases per 100,000 persons | Predominant lineage/variant | References |
| --- | --- | --- | --- | --- | --- |
| 1st | June 2020 | ≈ 199,400 | 37.0 - 40.7 | 1.1.54, B.1.1.56 & C.1 | [53̶ 55] |
| 2nd | Dec 2020 | ≈ 170,687 | 31.1 - 41.5 | Beta (B.1.351) |  |
| 3rd | July 2021 | ≈ 220,568 | 37.3 - 46.5 | Delta (B.1.617.2) |  |
